# Multiparametric Color Tendency Analysis (MCTA): A Method to Analyze Several Flow Cytometry Labelings Simultaneously

**DOI:** 10.3389/fbioe.2020.526814

**Published:** 2020-09-17

**Authors:** Andrea Henriques-Pons, Carine P. Beatrici, Juan Camilo Sánchez-Arcila, Fabricio Alves Barbosa da Silva

**Affiliations:** ^1^Laboratório de Inovações em Terapias, Ensino e Bioprodutos, Instituto Oswaldo Cruz, Fundação Oswaldo Cruz, Rio de Janeiro, Brazil; ^2^Scientific Computing Program, Fundação Oswaldo Cruz, Rio de Janeiro, Brazil; ^3^Department of Natural Sciences, University of California, Merced, Merced, CA, United States

**Keywords:** flow cytometry, multiparametric data analysis, software, method, t-SNE, MCTA

## Abstract

Despite the remarkable evolution of flow cytometers, fluorescent probes, and flow cytometry analysis software, most users still follow the same ways for data analysis. Conventional flow cytometry analysis relies on the creation of dot plot sequences, based on two fluorescence parameters at a time, to evidence phenotypically distinct populations. Thus, reaching conclusions about the biological characteristics of the samples is a fragmented and challenging process. We present here the MCTA (Multiparametric Color Tendency Analysis), a method for data analysis that considers multiple labelings simultaneously, extending and complementing conventional analysis. The MCTA method executes the background fluorescence exclusion, spillover compensation, and a user-defined gating strategy for subpopulation analysis. The results are then presented in conventional FSC x SSC dot plots with statistical data. For each event, the method converts each of the multiple fluorescence colors under analysis into a vector, with longer vectors being attributed to more intense labelings. Then, the MCTA generates a resultant vector, which is therefore mostly influenced by predominant labelings. The radial position of this resultant vector corresponds to a resultant color, making it easy to visualize phenotypic modulations among cellular subpopulations. Besides, it is a deterministic method that quickly assigns a resulting color to all events that obey the gating strategy, with no polymeric regions defined by the user or downsampling. The MCTA application generates a single dot plot showing all events in the FCS file, but a resultant color is attributed only to those that obey the gating strategy. Therefore, it can also help to evidence rare events or unpredicted subpopulations naturally excluded from the regions defined by the user. We believe that the MCTA method adds a new perspective over multiparametric flow cytometry analysis while evidencing modulations of molecular labeling profiles based on multiple fluorescences. Availability and implementation: The instructions for the MCTA application is freely available at https://github.com/flowcytometry/MCTA.

## Introduction

The flow cytometry technique offers quantitative fluorescence-based data, usually regarding cellular characteristics, at a rate of hundreds of events per second (Shapiro, 2004). Modern flow cytometers detect more than 15 parameters of fluorescence per event, evaluating cellular phenotype, viability, and proliferation; Ca^++^ levels; organelles activity; and much more. All technological advances of flow cytometry resulted in an enormous development in biology and medicine. Still, for reproducible results, all preliminary steps must be carefully planned, considering data generation, data pre-processing and quality control, visualization of results, and final data analysis (Saeys et al., 2016).

Despite the increasing complexity and evolution of multiparametric flow cytometry, conventional data analysis is still based on the evaluation of up to two fluorescence parameters at a time, relying on the creation of multiple dot plots (Mair et al., 2016). The gating strategy also obeys a logical hierarchical sequence of regions drawn manually, and users must reach an experimental conclusion after a fragmented analysis (de Oliveira et al., 2007; Cascabulho et al., 2016). Although flow cytometrists are used to this method, some disadvantages are the manual and imprecise definition of regions (gates), the underestimation and low visibility of rare events, and the fact that minor subpopulations outside the user-defined regions are not considered in the analysis.

To address the lack of exploratory analysis of flow cytometry data and the issues with reproducibility, several algorithms and computational tools have been developed (Pedreira et al., 2019). For instance, some dimensionality reduction algorithms used in flow cytomety data analysis are t-distributed stochastic neighbor embedding (t-SNE) (Toghi Eshghi et al., 2019) and Uniform Manifold Approximation (UMAP)^1^. Both are dimension reduction algorithms that favor the preservation of local distances over global distance. Other methods use clustering algorithms that show spanning trees as a result, like in Spanning-tree Progression Analysis of Density-normalized Events (SPADE) (Mair et al., 2016) and FlowSOM (Van Gassen et al., 2015). In these methods, the cells are represented by nodes connected with the neighbors in the high dimensional data. Therefore, the interconnected nodes are related to phenotypically similar cells, as shown by the CITRUS algorithm (Bruggner et al., 2014). Moreover, subpopulation detection in high-dimensional data can be analyzed using PhenoGraph (Levine et al., 2015). An excellent review of these and other complementary strategies can be found in Weber and Robinson (2016).

Typical workflows in computational tools include data transformation, normalization, filtering, manual or semi-automatic gating, and automatic clustering (Montante and Brinkman, 2019). However, only advanced users with programming skills will be able to go through the analysis process. For regular users, manual efforts remain standard practice, which has been mostly the same for decades, regardless of the commercial analysis software used. To address these issues, we present the MCTA (Multiparametric Color Tendency Analysis). This is an alternative exploratory method that analyzes multiple phenotypic markers simultaneously and evidences complex cellular profiles, different from the multi-step conventional analysis. The method excludes the background range of each channel for each event, according to negative controls, and spillover fluorescence is compensated. Moreover, the gating procedure for subpopulation analysis is done in a single step, including and excluding multiple chosen cellular markers according to the user rationale for cellular identification. Then, to show the phenotypic result, the algorithm attributes a base vector directly proportional to the labeling intensity of each fluorescence parameter to be analyzed, and a resultant vector is calculated for each event. According to the radial position of the resultant vector, a different resultant color is attributed to each event. Therefore, the resultant color is determined by the predominant labeling or labelings under analysis. In the MCTA analysis, biological modulations of experimental target molecules are easily visualized by different resultant colors, and the results are backed up by statistical analysis for data interpretation. Moreover, the MCTA method maps predominant phenotypic profiles on conventional morphology FSC-A vs. SSC-A dot plots, an option that standard multivariate algorithms do not offer.

## Computational Method

### Color Representation in the MCTA Method

**FIGURE 1:**
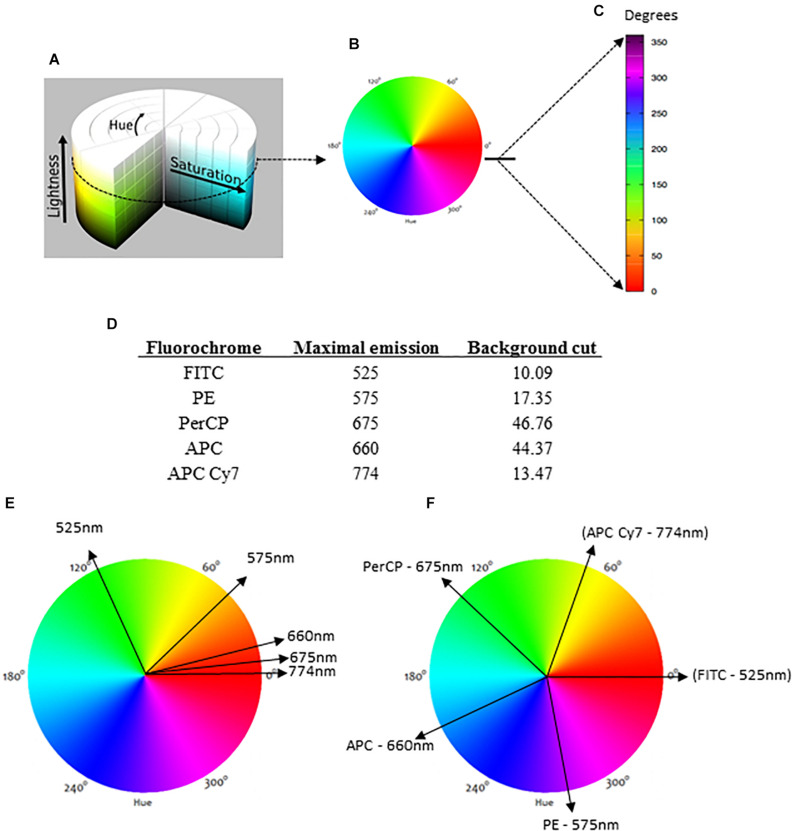
The HSL (hue, saturation, lightness) representation and the basis for data analysis. The conventional HSL representation is a cylinder **(A)**, but for flow cytometry analysis, we considered lightness as a constant factor for a bi-dimensional diagram **(B)**. For the representation next to the dot plots, a linear version of the bidimensional HSL was produced with reference degrees indicated **(C)**. For the analysis, each fluorochrome is indicated by its maximal emission of fluorescence, and the background (negative range) is required **(D)**. When each fluorochrome is represented in a circular bidimensional HSL diagram, different parameters with similar maximal emissions are closely indicated **(E)**. To avoid this overlapping color representation, our method automatically divides the HSL into equal parts **(F)**.

The color assignment adopted in this work is based on the HSL representation (Hu et al., 2014) (Figure 1A). HSL stands for hue, saturation, and lightness (or luminosity) and consists of a cylindrical-coordinate representation of points in an RGB (red, green, blue) color model. In this representation, the RGB coordinates are geometrically arranged in an attempt to be more intuitive (Figure 1A). Developed in the 1970s for computer graphics applications, the HSL is used today in color pickers, image-editing software, and less commonly in image analysis and computer vision (Tsai and Tseng, 2012).

In the general HSL representation, each color is a dot in a cylinder, the angle around the central vertical axis corresponds to “hue,” the distance from the axis corresponds to “saturation,” and the distance along the axis corresponds to “lightness” (Figure 1A). For flow cytometry, the HSL was chosen to represent fluorescence data because each color can be represented as an individual vector with a corresponding angular value. The proportional intensity of each labeling (fluorescence parameter) is represented by the saturation, with longer vectors representing more intense fluorescence labelings. For a bi-dimensional representation, we considered lightness as a constant factor (Figures 1A,B). Then, the spectrum was represented as a linear rule with the hue values (fluorescence colors) ranging from 0 to 360° or *0 rad* to *2π rad* (Figure 1C).

Once the MCTA method uses all labeling colors determined by the user simultaneously, each fluorescence parameter must be identified for the vectorial representation per event. For this, our method uses the maximal emission value of each fluorochrome used (Figure 1D) and projects a correspondent vector over the bi-dimensional HSL representation (Figure 1E). As many fluorochromes have similar or even the same maximal emission value, the MCTA method divides the spectrum (360°) into equal parts automatically (Figure 1F). Therefore, in the MCTA method, the color attributed to each fluorescence parameter does not correspond to the real emission of the fluorochrome (Figure 1F).

### Event Representation in the MCTA Method

The MCTA method computes a resultant vector to each event, using all labeling colors chosen at the same time. This resultant vector determines a resultant color observed in the HSL representation and will be most affected by the predominant labeling(s). This analysis, therefore, aims to show the tendency of labeling(s) with higher median fluorescence intensity (MFI) in each event. When comparing different experimental groups, such as uninfected and pathogen-infected mice, for example, this analysis will quickly show if there was a difference in the repertoire of mostly expressed molecules between samples, showing the cellular profile.

**FIGURE 2:**
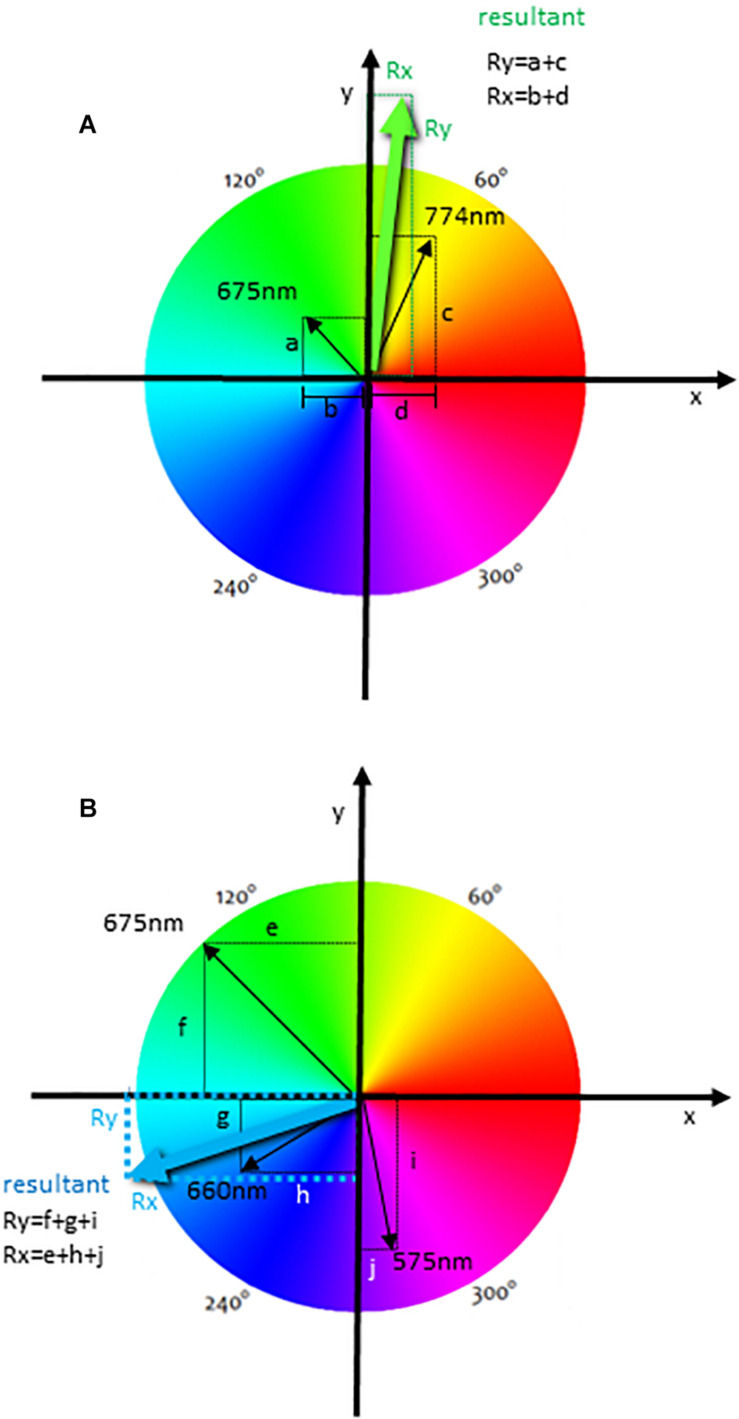
The calculation of a resultant vector using the HSL representation in flow cytometry analysis. The diagrams show one event with two labelings (maximal emissions are 675 and 774 nm) **(A)**, and with three labelings (maximal emissions of 575, 660, and 675 nm) **(B)** used for the resultant vector calculation. Black arrows indicate fluorescence labeling colors, and the length of these arrows is directly proportional to the intensity of each labeling. These vectors project segments over the X- and Y-axis, indicated by letters, and the resultant vector indicating the resultant color is calculated using all segments for each event. The resultant segments are indicated as Rx and Ry.

In Figure 2A, we illustrate the generation of the resultant vector using an event that was labeled with different fluorochromes, but only two of these parameters were used to generate the resultant vector. Therefore, the spectrum was divided into equal parts according to the number of fluorescences, but only two vectors were accounted for the resultant color. In this case, we represented the PerCP (maximal emission of 675 nm) and APC Cy7 (774 nm) (Figure 1D) labelings. The intensity of each labeling was proportionally represented by the length of each vector (represented in black) (Figure 2A). These vectors project segments over the axes X and Y, which are “b” and “a” for the fluorochrome 675 nm and “d” and “c” for the fluorochrome 774 nm (Figure 2A). The resultant vector was produced by the segment “d” minus “b” projected over the X-axis Rx (as the segment b is below zero) (Rx) and by the summon of the segments “a” and “c” projected over the Y-axis (Ry) (Figure 2A). The calculated segments Rx and Ry determined the resultant vector, indicating the color to be attributed to the event (Figure 2A). In another analysis, we illustrate the generation of the resultant vector using three of the experimental labelings (Figure 2B), which were 575, 660, and 675 nm (Figure 1D). In this case, the resultant vector was generated by the projection of the segments “e,” “h,” and “j” over the X-axis (Rx) and by the segments “f,” “g,” and “i” over the Y-axis (Ry). In Figure 2 we show that the resultant vector for each event was obtained as a function of the angle (hue) of each fluorescence vector and the intensity of individual color labelings.

Formally, for each wavelength L_j_ we associate one hue value h_j_, which is a degree on the color wheel and is directly related to the vector’s ${\vec{\text{v}}}_{\text{j}}$ angle. The j-th unit vector is given by:

(1) $${\overset{\wedge }{v}}_{j}=\cos\left(h_{j}\right)\overset{\wedge }{x}+\sin\left(h_{j}\right)\overset{\wedge }{y}$$

The resultant color is calculated considering all fluorescence channels that were chosen and their intensities (saturation) as bi-dimensional vectors. The final color of the event will be the vectorial sum of all fluorescence colors chosen, with the intensity of the fluorescence channel for each event *I*_*j*_ being taken into account. The resultant vector of the i-th event is given by:

(2) $${\vec{t}}_{i}=\sum_{j=1}^{n_{c}}I_{j}{\overset{\wedge }{v}}_{j}$$

where the I_*j*_ is the intensity (saturation) of the j-th channel of the event, *n*_*c*_ is the number of channels and *v*∧j is the filter unit vector.

### Configuration File

**FIGURE 3:**
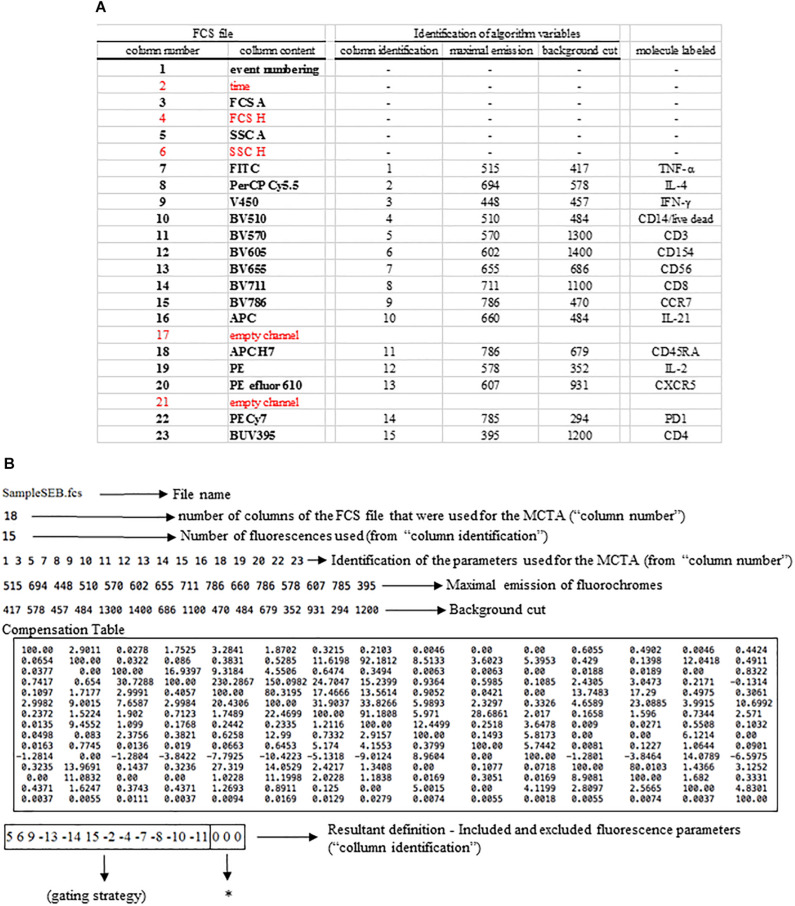
Logical and syntax rules for the configuration files. All flow cytometry files were organized in columns indicated here as “column number” and “column content” under “FCS file” **(A)**. All parameters shown in red were not used in the example depicted. For the production of the configuration file, the original parameters were renumbered, considering only the fluorescence colors, and all other parameters must be entered as indicated **(B)**. In the line identified as “resultant definition,” each inclusion and exclusion parameter is indicated, and the parameters used for the MCTA’s resultant vector are identified as zeros (**B**, asterisk).

The MCTA method follows required steps for any flow cytometry analysis, which are the compensation of spillover fluorescence (illustrated in Box 1) and the definition of fluorescence intensity thresholds (background) (Figure 1D). Here we present the MCTA analysis using two sources of FCS files. We used a simple five-color labeling experiment uploaded to the Github repository^2^ for a detailed description of the method. Then, to demonstrate the application of the algorithm, we used flow cytometry FCS files available in a public repository^3^. For the results section, we used data that evaluated T lymphocyte response after Staphylococcal enterotoxin B (SEB) stimulation (repository ID FR-FCM-ZZEC, 15 colors) and a panel that identifies human adaptive natural killer (NK) cells (repository ID FR-FCM-ZYY6, 13 colors). It is important to highlight that, for each flow cytometry analysis in the MCTA method, a configuration file is created by the user, applying specific logical and syntax rules depicted in Figure 3. This example details the in. dat file containing all required parameters, considering original columns of the acquired FCS file (Figure 3A – FCS file) and the fluorescence parameters that must be renumbered to feed the algorithm (Figure 3A – column identification).

**Box 1. Compensation factors for spillover fluorescences (https://github.com/flowcytometry/MCTA).**

**Table:** 

	**FITC**	**PE**	**PerCP**	**APC**	**APC Cy7**
FITC	100.00	1.40	0.00	0.00	0.00
PE	18.22	100.00	0.00	0.00	0.00
PerCP	2.80	15.42	100.0	0.00	0.00
APC	0.00	0.41	6.86	100.00	14.49
APC Cy7	0.00	0.00	0.00	6.54	100.00

The first step is the identification of the FCS file under analysis (file name), which is SampleSEB.FCS, in this example (Figure 3B). Then, the user enters the total number of parameters of the FCS file and the ones that will be used in the MCTA method. In the example used in Figure 3B for lymphoid cells response against SEB, the FCS file had 23 columns (Figure 3A); however, only 18 columns were considered. Five columns (identified in red) were not used (time, FCS H, SSC H, and two empty channels) (Figure 3B). The next step is the definition of the total number of fluorescence parameters employed by the MCTA method. In this case, we used 15 (Figure 3B), which are the renumbered fluorescence parameters shown in the “column identification” (Figure 3A). The individual identification of what columns will be used is then entered. Note that the parameters that are shown in red (Figure 3A) were not included (columns 2, 4, 6, 17, and 21) (Figure 3B). As indicated before, the values of maximal emission of fluorochromes, background (negative range of fluorescence intensity per channel), and compensation factors are used in the MCTA method (Figure 3B). These values can be obtained from any flow cytometry analysis software and, in this case, were obtained using FlowJo version 10. Finally, in Figure 3A, we see that the renumbered fluorescence channels identified in the column “column identification” represent the labeling of the cellular markers identified in the column “molecule labeled.”

**FIGURE 4:**
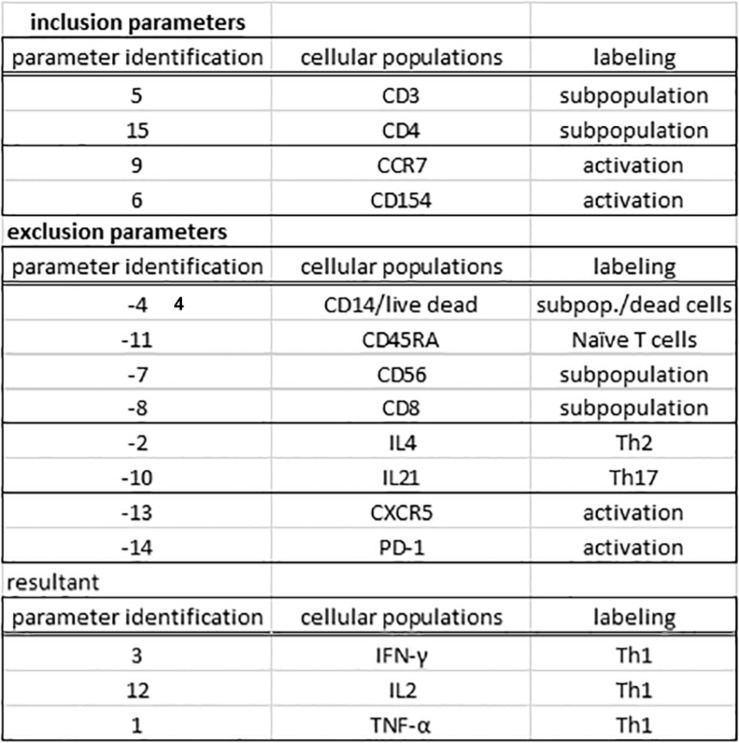
The gating procedure in the MCTA method. The gating process in the MCTA method is based on the definition of inclusion parameters numerically identified in “parameter identification.” In this case, only events positively labeled for CD3, CD4, CCR7, and CD154, identified as parameters 5, 15, 9, and 6 indicated under “cellular subpopulation” and “labeling” will be used in the gating strategy. The exclusion parameters used in this example were identified as –4, –11, –7, –8, to exclude cellular subpopulations and naúve T cells, –2 and –10, to exclude Th2 and Th17 cells, and –13 and –14 as activation T cell markers expressed on few events. The resultant vector was calculated using only the parameters 3, 12, and 1.

One essential step for any flow cytometry analysis is the gating process, which is the definition of cellular subpopulations of interest. The gating procedure in the MCTA method is done including and excluding events that are positive for given cellular markers, and this process is illustrated in Figure 3B – resultant definition; gating strategy, Figure 4; and Supplementary Figure 1. In the example shown in Figures 3B, 4, the analysis was done only in the events that were positive for CD3, CD4, CCR7, and CD154 (parameters 5, 6, 9, and 15), illustrating that the analysis was done in activated CD4^+^ T lymphocytes (Figure 4). The indication of exclusion parameters means that the MCTA method will not calculate the resultant color in any of the events that are positive for any of the exclusion parameters. In this case, there will be no resultant vector calculation for events identified as monocytes (CD14^+^, parameter 4), dead cells (positive events for Live Dead labeling, also parameter 4), naïve T cells (CD45RA^+^, parameter 11), NK or NKT cells (CD56^+^, parameter 7), CD8^+^ T lymphocytes (parameter 8), or T cells expressing IL-4 or IL-21 (parameters 2 and 10, respectively). In this example, although CXCR5 (parameter 13) and PD-1 (parameter 14) are molecules associated with T lymphocyte activation, very few events were expressing either molecule, and we decided to exclude both. In the MCTA method, the exclusion parameters are indicated by the minus sign (Figure 3B – resultant definition, gating strategy), and the events that are labeled with any of the exclusion parameters will be represented in black. Finally, after the gating procedure, the syntax to represent the parameter(s) used for the generation of the resultant color is “zero” (Figure 3B asterisk – resultant definition). In this particular case, IFN-γ, IL-2, and TNF-α were the parameters used for the MCTA analysis, indicated in the “column identification” as parameter 3, 12, and 1 (Figures 3A, 4). However, in the configuration file, all three parameters are represented as zeros (Figure 3B – resultant definition; asterisk). In biological terms, this analysis targets the identification of Th1-responding activated CD4^+^ T lymphocytes.

## Results

### The MCTA Analysis

**FIGURE 5:**
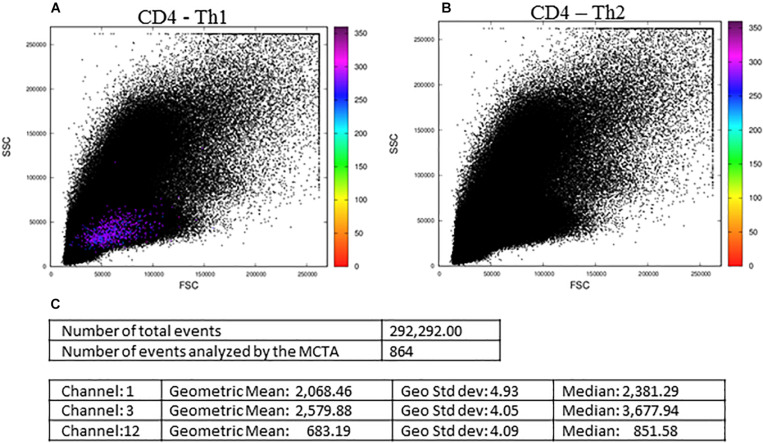
The MCTA application and resultant dot plots: the MCTA analysis was done in Th1 **(A)** and Th2 **(B)** CD4^+^ T lymphocytes obtained from healthy human donors and stimulated *in vitro* with Staphylococcal enterotoxin B (SEB). The MCTA gating strategy considered the events positive for CD3, CD4, CCR7, and CD154 labeling, and colored events correspond to the calculation of a resultant vector (or color) based on the labeling of IFN-γ, IL-2, and TNF-α **(A)** or IL-4 **(B)**. The MCTA statistical analysis is shown **(C)** for the file represented in **(A)**. These FCS files were obtained from the repository (https://flowrepository.org/public_experiment_ representations) ID FR-FCM-ZZEC.

In Figure 5, we show the final result of the MCTA analysis, where SEB-activated CD4^+^ T lymphocytes, according to the gating strategy, are represented as colored events in the FSC x SSC dot plot. The color of each event in Figure 5A was attributed according to predominant labeling(s) of the Th1 cytokines selected for the analysis (Figure 4). When the configuration file was set up to evaluate Th2 (IL-4-producing) CD4^+^ T lymphocytes (Figure 5B), the analysis considered the inclusion and exclusion parameters shown in Supplementary Figure 1, and we observed no colored events. This result means that there were no stimulated CD4^+^ T cells positive for IL-4 labeling.

At this point, it was clear that the MCTA method was feasible and able to evidence a different pattern of results, showing the combination of multiple labelings for a phenotypic profile of gated subpopulations. However, it was still necessary to identify what parameters mostly influenced the resultant color observed and, for this, we added the statistical analysis. We then illustrated in Figure 5C, the statistical analysis of the data shown in Figure 5A. The MCTA method calculates conventional statistics that are most useful in flow cytometry analysis. It indicates the total number of events in the file and, regarding the colored events, the number of events that met the gating strategy, geometric mean, geometric standard deviation, and median (Figure 5C). The statistical analysis in the MCTA method is not based on the original data of the FCS file; it uses the processed data matrix created after the subtraction of the background and spillover compensation. Therefore, the user can visualize if only one or more fluorescence parameters were predominantly labeled, and what parameter(s) mostly influenced the resultant vector (the resultant color) (Figure 5C).

**FIGURE 6:**
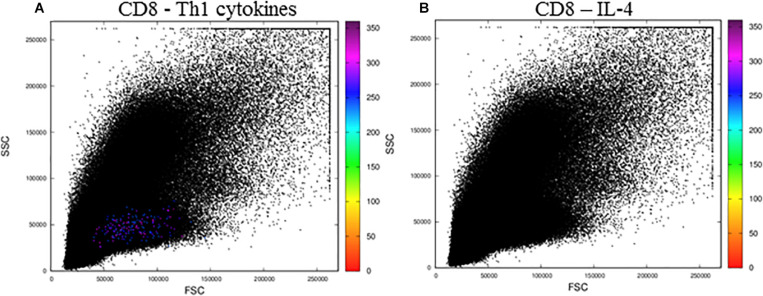
The MCTA application for the analysis of CD8^+^ T lymphocytes stimulated by SEB. The MCTA analysis was done in Th1 **(A)** or IL-4^+^ **(B)** CD8^+^ T lymphocytes obtained from healthy human donors and stimulated *in vitro* with Staphylococcal enterotoxin B (SEB). Colored events correspond to the calculation of a resultant vector (or color) based on the labeling of IFN-γ, IL-2, and TNF-α **(A)** or IL-4 **(B)** only in the events that are positive for CD3, CD8, CCR7, and CD154. These FCS files were obtained from the repository (https://flowrepository.org/public_experiment_representations) ID FR-FCM-ZZEC.

When we analyzed activated CD8^+^ T lymphocytes (Figure 6), we observed resultant color calculations only for cells producing Th1 cytokines (Figure 6A), and no cells produced IL-4 (Figure 6B). Moreover, when we analyzed the files obtained from the public repository corresponding to control cells incubated with DMSO, we observed no CD4 or CD8 T cells expressing any of the cytokines tested, as no resultant colors were generated (Supplementary Figure 2).

### Analysis of Rare Populations in the MCTA Method

The MCTA method has important advantages over conventional analysis and other computational tools, such as the quick analysis of many fluorescence parameters simultaneously. Moreover, the resultant vector (color) is shown per event on conventional morphology dot plots, familiar to all flow cytometry users, and the gating strategy is not influenced by regions defined by the user. One advantage is especially important in the MCTA method, which is the fact that all events are shown in the dot plot, colored or not. In conventional flow cytometry analysis, a sequence of manual regions defines the events that will be analyzed. Therefore, minor or unpredicted subpopulations outside the gates defined by the user are automatically excluded from the analysis. In the MCTA method, however, this is not an issue, as all events that obey the gating strategy are shown as colored events, including the ones that would not be in expected drawn regions.

**FIGURE 7:**
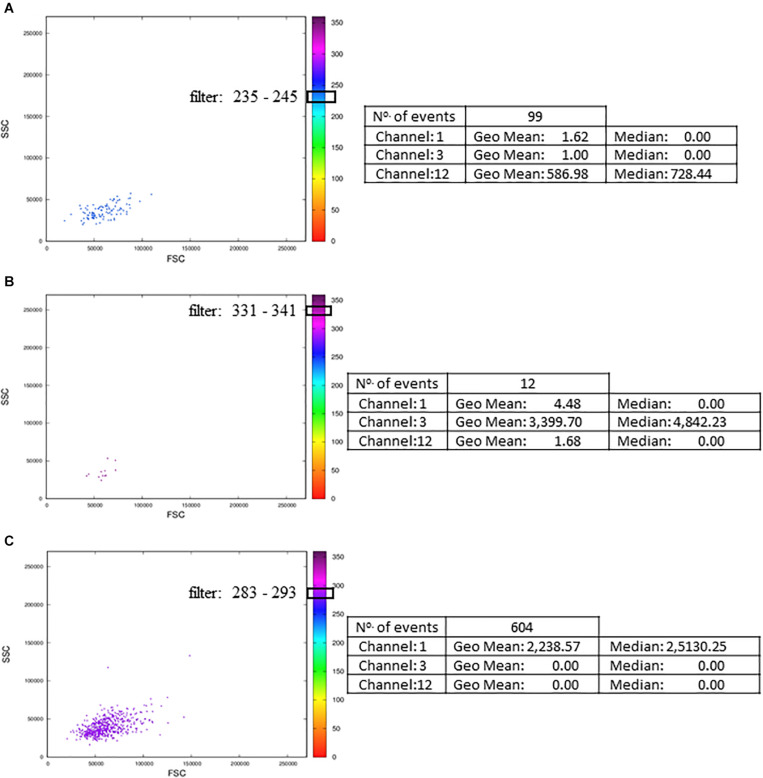
Resultant Plot With Filter: for the Resultant Plot With Filter, the user must define a range of degrees to apply to the MCTA analysis, and these ranges are graphically represented here as block boxes over the degree scale. The analysis was done in Th1 CD4^+^ T lymphocyte obtained from healthy human donors and stimulated *in vitro* with Staphylococcal enterotoxin B (SEB). The gating strategy considered the events positive for CD3, CD4, CCR7, and CD154 labeling. The resultant vector was calculated based on the labeling of IFN-γ, IL-2, and TNF-α, and the statistical analysis of each range is shown. The examples considered only events with resultant colors in the range of 235 to 245° (**A**, the color range of IL-2), 331 to 341° (**B**, the color range of IFN-γ), and 283 to 293° (**C**, the color range of TNF-α). These FCS files were obtained from the repository (https://flowrepository.org/public_experiment_representations) ID FR-FCM-ZZEC.

Although the MCTA method can identify rare events, it can be challenging to visualize these few colored events in files that have many events; then, we created one interactive dot plot as an additional tool. In this case, the user can determine a narrow range of degrees to observe only the events displayed in that range of resultant colors. This dot plot was named Resultant Plot With Filter, and it excludes uncolored events and events outside the range selected (Figures 7A–C). Then, the statistical analysis corresponds only to those events that were included in the range defined (Figure 7). In this example, the ranges corresponded to 10 degrees, but the user delimitates the range according to individual results. It is important to highlight that for rare events identification, as in any flow cytometry analysis method, the number of acquired events will be critical for data visualization.

**FIGURE 8:**
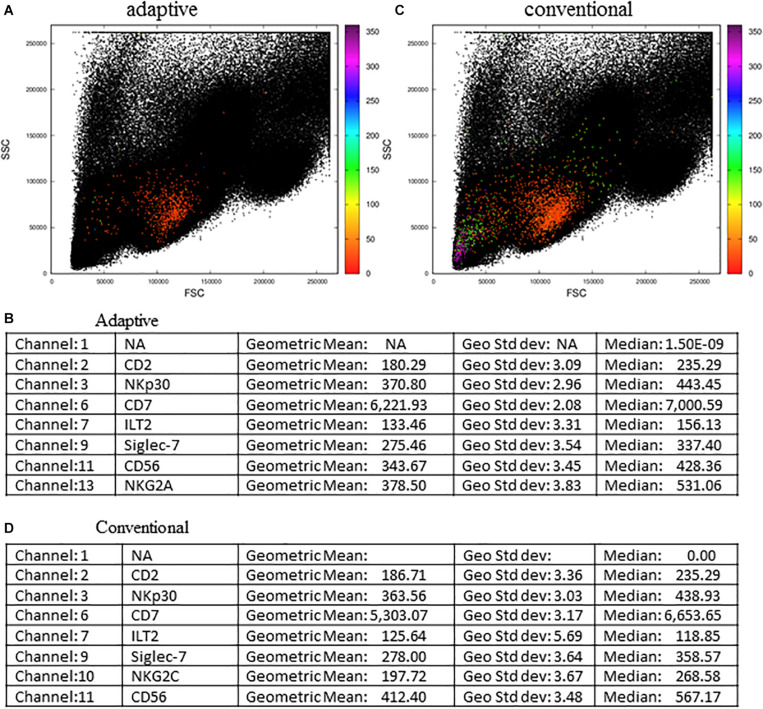
Adaptive and conventional NK cells analysis using the MCTA method. The expression of NKG2C was the inclusion parameter for the analysis of adaptive NK cells **(A)**, and the expression of NKG2A **(C)** was used for the study of conventional NK cells. Statistical analysis for adaptive NK cells is shown in **(B)** and for conventional cells is shown in **(D)**. These FCS files were obtained from the repository (https://flowrepository.org/public_experiment_representations) repository ID FR-FCM-ZYY6.

We further challenged the MCTA method using other files downloaded from the repository, and the next experiment evaluated the phenotype of adaptive and conventional NK cells (Figure 8). For this analysis, we arranged the fluorescence colors (parameters) as indicated in the Supplementary Figures 3A–C, and it shows that the original FCS file had 17 parameters (Supplementary Figure 3B). For the analysis of adaptive NK cells, we defined as exclusion parameters the events corresponding to dead cells, monocytes (CD14^+^) and B lymphocytes (CD19^+^) (all in parameter 4), T lymphocytes (CD3^+^ events, parameter 12), and events positive for an adaptive NK cells marker that labeled few events (CD57^+^ events, parameter 5) (Supplementary Figure 3B). The inclusion parameter corresponded to the expression of only NKG2C (adaptive NK cells, parameter 10) (Supplementary Figure 3B – indicated by the box “Resultant definition for adaptive NK cells”) and for the MCTA analysis, we used a different proposal than the one used by the authors. For the resultant color calculation, we selected the channels that corresponded to two available columns and the expression of CD2, NKp30, CD7, ILT2, Siglec-7, CD56, and NKG2A (parameters 1, 8, 2, 3, 6, 7, 8, 9, 11, and 13, respectively) (Supplementary Figures 3A,B). Although we included the available parameters 1 and 8 in the analysis, these variables do not affect the resultant color, as predominant labelings mostly influence the result.

As a result of adaptive NK cells, we observed most events as orange cells (Figure 8A) and the statistical analysis, considering geometric mean and median, showed that parameter 6 (CD7 labeling) was the single predominant labeling that accounted for the resultant color in the MCTA analysis (Figure 8B).

For conventional NK cells, we used the exclusion parameters shown in Supplementary Figure 3C (parameters 4, 12, and 5), and the inclusion parameter was parameter 13 (NKG2A). The MCTA analysis revealed different subpopulations of NKG2A^+^ cells, with orange, green, and violet as resultant colors (Figure 8C). This means that subpopulations of NKG2A^+^ cells had different predominant markers, which would be difficult to observe in conventional analysis. In this particular case, despite the heterogeneous subpopulations (Figure 8C), the statistical analysis showed the parameter (channel) 6 as the predominant labeling channel (Figure 8D). It happens because the MCTA analysis is done at the event level, and the statistics give populational results, as any flow cytometry analysis. To find the predominant labeling(s) that generated the different resultant colors (subpopulations), the Resultant Plot With Filter is once more the solution. In this case, the user determines a range of degrees that selects only the events shown in a given color, either orange, green, or violet. Therefore, the statistics will show the predominant labeling(s) for each subpopulation of cells. As shown in Figure 7, the statistics will be restricted to each range of degrees for each subpopulation, and the user will be able to identify what molecule or molecules mostly contributed to each resultant color.

## Discussion

During the past few decades, we witnessed the development of new software and tools for the analysis of increasingly complex results obtained by flow cytometry. However, most users still follow the same analysis strategies, typically based on the definition of sequences of manual gates on multiple dot plots that show two parameters at a time. Although cytometrists are familiar with this fragmented process, it has many disadvantages that affect data reproducibility and accuracy.

Here we propose an automated strategy to explore the diversity of cells in flow cytometry data. To the best of our knowledge, the MCTA method is the first algorithm that analyzes multiple labelings simultaneously and maps the results in FSC-A x SSC-A morphology gates, extending the conventional analysis. The process is accessible for average users and can quickly show the resultant color based on multiple fluorescence labelings per sample. However, at this point, we present the rationale of the MCTA method, which is intended to be included in software or packages for flow cytometry analysis in user-friendly interfaces. It is our primary goal to offer users a different perspective on the complexity of their results. This method allows the observation of biological phenomena that could not be identified if any other tool were used. In this way, the application of the MCTA method can guide subsequent analysis choices, allowing as much information as possible to be extracted from biological samples. Moreover, the results are reproducible among collaborators, as long as using the same files and applying the same background and compensation values.

Here, we used FCS files downloaded from a public repository and challenged the method using two sets of data. These experiments employed 13 and 15 fluorescence parameters that were used for the gating strategy and the calculation of the resultant color. In these examples, the resultant color was obtained based on single labeling (IL-4), three labeling colors, or nine colors. Even if the number of parameters used to generate the resultant vector exceeds nine colors, this does not impose a limitation on the MCTA application. Because of the statistical analysis, the users will always identify what marker or markers mainly influenced the resultant vector generated for data interpretation. The Resultant Plot With Filter is another tool developed to help the users in the interpretation of the results, which is never exclusively based on the visual identification of the color of the events.

One essential aspect of the MCTA method is its determinism. Several flow cytometry methods for data analysis use stochastic algorithms for dimensionality reduction, which may require great processing capacity. To address this problem, it is common to reduce the number of events analyzed to keep running times acceptable (Saeys et al., 2016). Moreover, stochastic methods like t-SNE or UMAP produce different results for each run on the same dataset; therefore, the users should run the algorithm multiple times using the same data to analyze variability and prevailing trends. Furthermore, stochastic methods are typically able to process only a few tens of thousands of events per run, even when implementing additional techniques such as downsampling, hierarchical clustering, or dimensionality reduction. Another obstacle in the use of more complex dimensionality reduction techniques is the setting of the parameters to run the algorithms. Frequently, this inherent complexity leads the users to employ default settings to run their analysis due to the lack of knowledge to change these parameters. In the case of t-SNE for example, the user has to choose values for the perplexity parameter, whose typical values vary between 5 and 50. As a stochastic method, even when using the same perplexity parameter value, the result will variate when comparing different runs. Moreover, it was recently published that t-SNE can erroneously indicate clusters for homogeneously distributed data, suggesting the wrong number of subgroups or projecting data points that belong to the same subset, as if they belong to different subgroups (Lötsch and Ultsch, 2019).

A deterministic method, like the MCTA algorithm, requires only one run on a dataset and does not demand additional aggregation steps. Furthermore, the complexity of the algorithm described in this work is O(N.*nc*), where N is the number of events, and *nc* is the number of channels. Typically *nc* is fixed (for a specific flow cytometer) and *nc* < < N; therefore, the complexity can be approximated by O(N).

Our method can also be easily adapted to explore multiple cores/nodes in parallel, taking full advantage of modern multi-core processors in highly scalable implementations. Indeed, the method proposed here is very efficient computationally, considering both execution time and memory requirements. When we compared the processing times of MCTA vs. t-SNE to generate Figure 8 using all events, we observed that MCTA spent up to half a minute processing the whole data set. In contrast, the t-SNE analysis required more than 6 h using an average computer (Mac Book pro, 16gb RAM, 2.3 GHz Intel Core i5) and up to 2.5 h in a computational cluster. All these characteristics make the MCTA an ideal method to rapidly evaluate specific questions about cellular phenotype or function. Using the same computer mentioned, it was impossible to run a UMAP dimension reduction technique due to computational restrictions. To date, when we performed a downsample of 15,000 events, we noted a dramatic reduction in the events gated in the MCTA analysis, showing the importance of analyzing the whole set of data. Although downsampling strategies are frequently applied to the data before using dimensional reduction techniques, our observation suggests that data might be lost during downsampling to run t-SNE or UMAP algorithms. Therefore, the identification of rare populations can be impossible.

Although the MCTA method is based on the calculation of a resultant vector according to multiple labelings, events with extreme artifactual fluorescent signals (very high or very low MFI), will not alter the tendency result of the population under analysis. This is true because the MCTA method calculates a resultant color for each event, and extreme artefactual signals will affect only the event itself, not the population under analysis. Moreover, the MCTA method is not indicated to substitute conventional analysis; it is proposed as a new way to show complex phenotypic profiles, complementing and extending conventional analysis. We believe that conventional flow cytometry data analysis, combined with other appropriate computational tools and methods, will help to identify and better describe biological phenomena, leading to more accurate, complete, and reproducible data.

We consider that the MCTA analysis offers results that are not directly comparable with traditional dimensionality reduction techniques as t-SNE and UMAP. In the MCTA method, the analysis is oriented to a specific set of data through the inclusion and exclusion of markers in the gating strategy. On the other hand, t-SNE and UMAP generally use the whole set of fluorochromes available in the data set.

Finally, we believe that the MCTA method can be integrated as a new functionality into flow cytometry analysis software, allowing complementary views and comparisons with well-established methods like t-SNE and UMAP and conventional fragmented analysis.

## Data Availability Statement

Publicly available datasets were analyzed in this study. This data can be found here: https://flowrepository.org/public_experiment_representations, ID: FR-FCM-ZZEC and https://flowrepository.org/public_experiment_representations repository, ID: FR-FCM-ZYY6.

## Ethics Statement

The animal study was reviewed and approved by A Comissão de Ética no Uso de Animais do Instituto Oswaldo Cruz (CEUA-IOC), certificate number: L006-2015.

## Author Contributions

CB proposed the HSL representation for the analysis of multiple labelings and wrote the initial version of the code. FS wrote the codes for the generation of dot plots and all statistical representation, made the functionality tests, and assisted in the writing of the text. AH-P did all biological tests and interpretation of MCTA results, wrote the article, and guided the MCTA method implementation according to biological parameters. JS-A reviewed the MCTA code and compared it with other computational tools, and helped writing the manuscript. All authors contributed to the article and approved the submitted version.

## Conflict of Interest

The authors declare that the research was conducted in the absence of any commercial or financial relationships that could be construed as a potential conflict of interest.

## Footnotes

1 https://arxiv.org/abs/1802.03426

2 https://github.com/flowcytometry/MCTA

3 https://flowrepository.org/public_experiment_representations

## References

[B1] Bruggner R. V. Bodenmiller B. Dill D. L. Tibshirani R. J. Nolan G. P. (2014). Automated identification of stratifying signatures in cellular subpopulations. *Proc. Natl. Acad. Sci. U.S.A.* 111 E2770–E2777. 24979804 10.1073/pnas.1408792111 PMC4084463

[B2] Cascabulho C. M. Beghini D. G. Meuser-Batista M. Penido C. Henriques-Pons A. (2016). Chemotaxis and immunoregulatory function of cardiac γδ T cells in dystrophin-deficient mice. *J. Immunol.* 197 3531–3544. 10.4049/jimmunol.1600335 27707996

[B3] de Oliveira G. M. Diniz R. L. Batista W. M. Batista M. Bani Correa C. de Araújo-Jorge T. C. (2007). Fas ligand-dependent inflammatory regulation in acute myocarditis induced by *Trypanosoma cruzi* infection. *Am. J. Pathol.* 171 79–86. 10.2353/ajpath.2007.060643 17591955 PMC1941608

[B4] Hu G. Pan Z. Zhang M. Chen D. Yang W. Chen J. (2014). An interactive method for generating harmonious color schemes. *Color Res. Appl.* 39, 70–78. 10.1002/col.21762

[B5] Levine J. H. Simonds E. F. Bendall S. C. Davis K. L. Amir E. A. Tadmor M. D. (2015). Data-driven phenotypic dissection of AML reveals progenitor-like cells that correlate with prognosis. *Cell* 162 184–197. 10.1016/j.cell.2015.05.047 26095251 PMC4508757

[B6] Lötsch J. Ultsch A. (2019). Current projection methods-induced biases at subgroup detection for machine-learning based data-analysis of biomedical data. *Int. J. Mol. Sci.* 21:79. 10.3390/ijms21010079 31861946 PMC6982269

[B7] Mair F. Hartmann F. J. Mrdjen D. Tosevski V. Krieg C. Becher B. (2016). The end of gating? An introduction to automated analysis of high dimensional cytometry data. *Eur. J. Immunol.* 46 34–43. 10.1002/eji.201545774 26548301

[B8] Montante S. M. Brinkman R. R. (2019). Flow cytometry data analysis: recent tools and algorithms. *Int. J. Lab. Hematol.* 41(Suppl. 1), 56–62. 10.1111/ijlh.13016 31069980

[B9] Pedreira C. E. Costa E. S. D. Lecrevise Q. Grigore G. Fluxa R. Verde J. (2019). From big flow cytometry datasets to smart diagnostic strategies: the EuroFlow approach. *J. Immunol. Methods* 475:112631. 10.1016/j.jim.2019.07.003 31306640

[B10] Saeys Y. Van Gassen S. Lambrecht B. N. (2016). Computational flow cytometry: helping to make sense of high-dimensional immunology data. *Nat. Rev. Immunol.* 16 449–462. 10.1038/nri.2016.56 27320317

[B11] Shapiro H. M. (2004). The evolution of cytometers. *Cytomet. A* 58 13–20. 10.1002/cyto.a.10111 14994215

[B12] Toghi Eshghi S. Au-Yeung A. Takahashi C. Bolen C. R. Nyachienga M. N. Lear S. P. (2019). Quantitative comparison of conventional and t-SNE-guided gating analyses. *Front. Immunol.* 10:1194. 10.3389/fimmu.2019.01194 31231371 PMC6560168

[B13] Tsai S.-H. Tseng Y.-H. (2012). A novel color detection method based on HSL color space for robotic soccer competition. *Comput. Math. Appl.* 64, 1291–1300. 10.1016/j.camwa.2012.03.073

[B14] Van Gassen S. Callebaut B. Van Helden M. J. Lambrecht B. N. Demeester P. Dhaene T. (2015). FlowSOM: using self-organizing maps for visualization and interpretation of cytometry data. *Cytomet. A* 87 636–645. 10.1002/cyto.a.22625 25573116

[B15] Weber L. M. Robinson M. D. (2016). Comparison of clustering methods for high-dimensional single-cell flow and mass cytometry data. *Cytomet. A* 89 1084–1096. 10.1002/cyto.a.23030 27992111

