# Supplementary figures and images for: Multiparametric Color Tendency Analysis (MCTA): A Method to Analyze Several Flow Cytometry Labelings Simultaneously
Source: Front Bioeng Biotechnol. 2020 Sep 17;8:526814. doi: 10.3389/fbioe.2020.526814 (PMC7527824; doi:10.3389/fbioe.2020.526814)

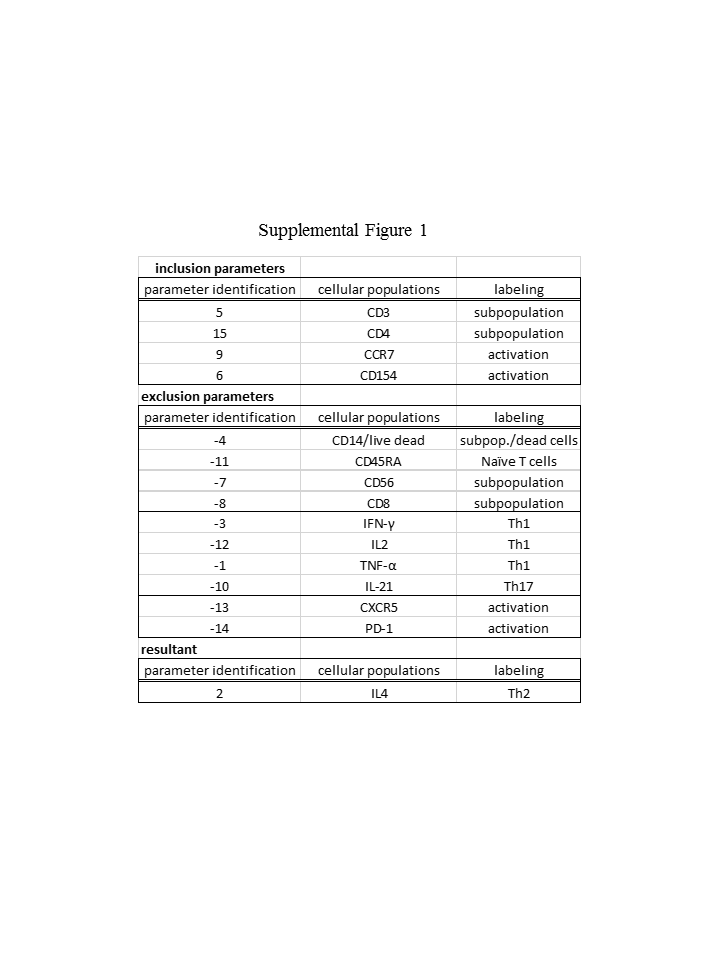

Supplement: Supplementary file 1 [file Image_1.TIF]

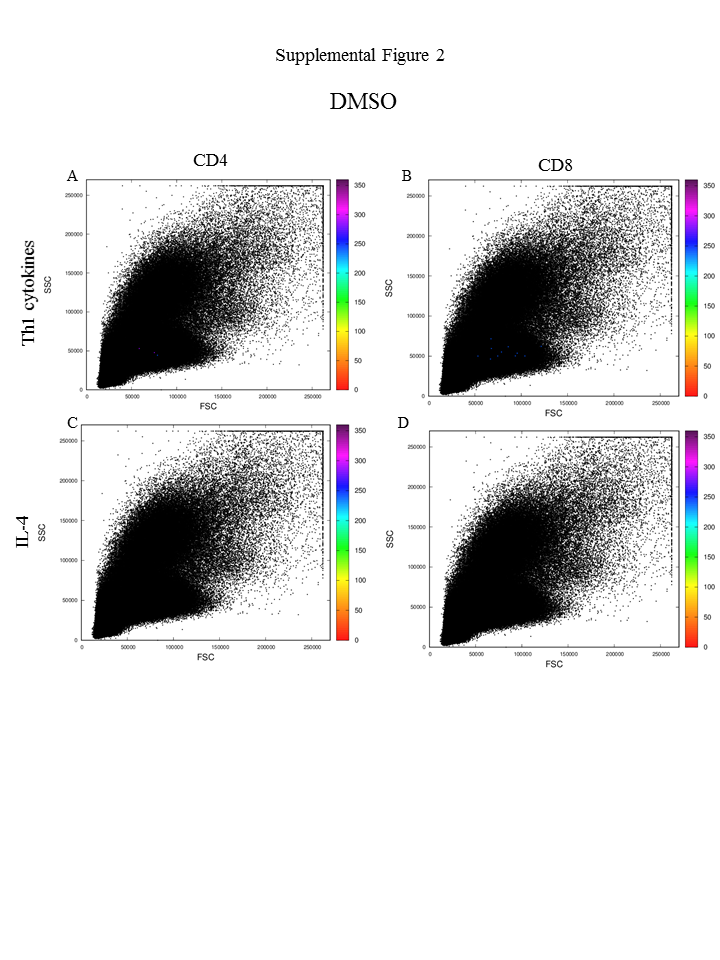

Supplement: Supplementary file 2 [file Image_2.TIF]

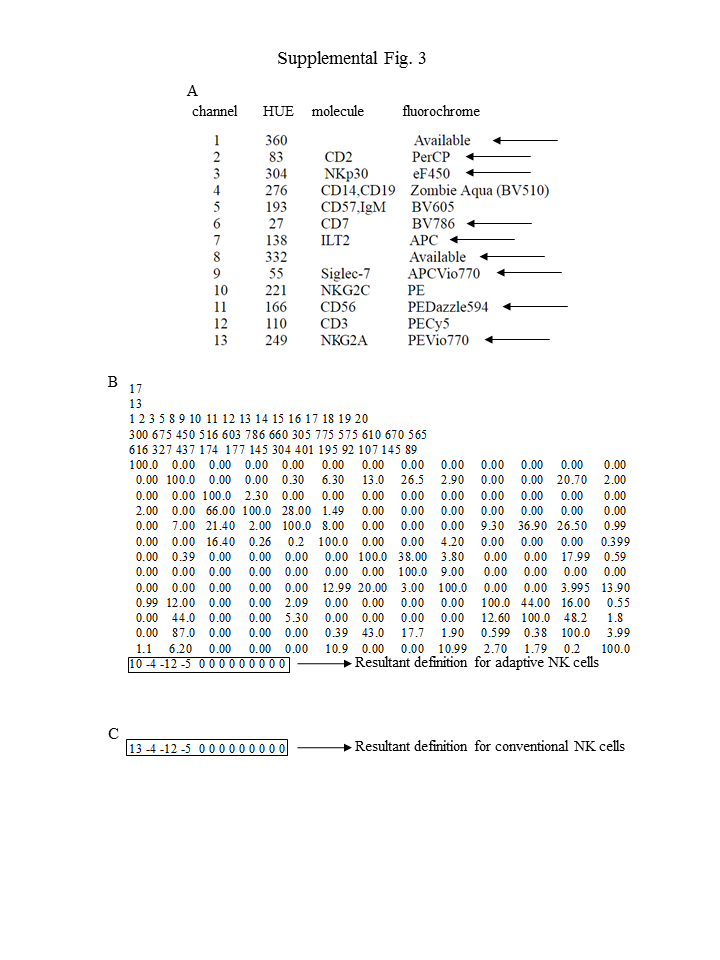

Supplement: Supplementary file 3 [file Image_3.TIF]
